# Barriers to access, transition and progression of Widening Participation students in UK Medical Schools: The students’ perspective

**DOI:** 10.15694/mep.2021.000132.1

**Published:** 2021-05-17

**Authors:** Nana Sartania, Louise Alldridge, Clare Ray

**Affiliations:** 1 University of Glasgow; 2 University of Plymouth; 3 University of Birmingham

**Keywords:** Widening Access, Admissions to medicine, Transition to university, Barriers to integration, Social, Economic and Cultural capital

## Abstract

This article was migrated. The article was marked as recommended.

Much attention is being given to the implementation of fair selection criteria for access to Medical Schools in the UK, in order to address an imbalance in social class representation in the medical professions. Largely overlooked however, are the disadvantages faced by potential applicants both before and after selection by the Medical Schools. Here, we explore the nature of the barriers - both real and perceived - to accessing, transitioning and progressing in the medical education system as experienced by 125 current students at three UK Medical Schools. An online survey was conducted and responses to open and closed questions were categorised on the basis of the number of Widening Participation (WP) flags (e.g. index of multiple-deprivation, parental education and school Higher Education participation rate). The results show that differences in economic, social and cultural capital that students acquire through their background impact on their chances of admission to medical schools, highlighting issues such as access to finance and privileged knowledge, with school support and work experience opportunities being less available to WP students. Equally, these students found the transition to Medical School harder, and highlighted a lack of peers from similar backgrounds, a perception of being less well prepared academically and generally finding it difficult to fit in. Money worries and having to work for extra income, exacerbated the feeling of not fitting in, by losing out on key extracurricular activities. The data presented stress the importance of early support of (potential) WP students in secondary schools, and of a strong support network throughout their medical education.

## Introduction

Social mobility is increasingly relevant in the UK, where there is an ever-rising income inequality and an unchanged overall measure of inequality, i.e. a Gini coefficient of 0.34, since the late 1980s ( Bourquin *et al.,* 2019; Milburn, 2009 and 2013), compared to 0.25-0.29 for most EU countries ( https://data.worldbank.org/indicator/SI.POV.GINI). Independent reviews on social mobility and child poverty ( Milburn, 2009; 2012; 2013) highlighted the need for fair access to Higher Education (HE) and noted that ‘medicine lags behind the other professions’ in this regard. Issues related to class and access to medicine were highlighted, stating that the vast majority of accepted medical school applicants came from the top three socio-economic classes, with just 7% from the bottom three classes, establishing a clear rationale for widening participation (WP) in Medicine.

WP is the process by which underrepresented groups are encouraged to apply to HE ( Cleland *et al.,* 2014; Patterson and Price, 2017), but it must also encompass strategies to support the retention and progression of students throughout their undergraduate studies and beyond ( HEA, 2015). Patients are more willing to access health care when they feel supported and understood by doctors from their own social backgrounds ( Girotti, Park and Tekian, 2015) and medical students from lower socioeconomic backgrounds tend to work in these areas after graduation ( Garlick and Brown, 2008).

A successful strategy to widen participation of underrepresented groups requires an in-depth understanding of the unique barriers students encounter when engaging in professional courses like Medicine. The barriers are complex and surpass academic attainment and non-cognitive attributes. Different sets of social, cultural and economic capital are acquired by people from different backgrounds, which allow them to be successful in certain ‘fields’ ( Bourdieu, 1986). Wealth, social connections, symbolic cultural cues and dispositions contribute to Bourdieu’s ‘habitus’, which is inextricably linked to a classed system and 2-tier primary and secondary education. To successfully access medicine, a student’s habitus must match what is deemed important by medical schools’ selectors, which in themselves are also linked to a habitus acquired through class and school type. It is possible that this mismatch impedes retention of students from a WP background through a reduced sense of belonging and ‘fitting in’.

Although entry rates into HE from students in deprived areas have improved for many degrees through introduction of various WP programmes, non-continuation rates are rising in disadvantaged students (Higher Education Statistics Agency, 2016). Anecdotally, the barriers remain *after* these students enter Medical School and this issue is yet to be addressed.

This paper looks at those barriers, real and perceived, as experienced by current medical students at three very different medical schools, Glasgow, Birmingham and Peninsula. Each school deploys different selection methods, WP strategies, and learning and assessment approaches, providing a broad student perspective that allowed us to identify cross-cutting themes and issues.

## Methods

Ethical approval for the project was granted by the Universities of Birmingham (ERN17-0802), Glasgow (200140188) and Plymouth (17/19-986). Students were asked to take part in an online survey (JISC/Survey Monkey) with closed and open questions ( *Supplementary File 1*), which gathered quantitative and qualitative data from a sample of 125 responding students.

Participants were recruited from a sub-population of students from the Widening Access to Medicine Student Society (WAMSS) at Glasgow and Peninsula Medical Schools and those who had participated in outreach programmes: Reach (Glasgow), Routes to the Professions: Medicine (Birmingham) and Pathways to Healthcare Professions (Peninsula). Students were informed of the study design by email. Informed consent was obtained after which the survey link and participant information sheet were distributed electronically.

Students were asked to provide data on their demographics and reflect on their experience of transition to university. The demographic data (the lowest two categories of Scottish Index of Multiple Deprivation (SIMD)/Participation in Local Areas (POLAR); attendance at a low progression school; receipt of free school meals and/or educational maintenance grant; first in family to attend university; experience of local authority care) were used to categorise students into four groups based on the number of WP flags they had (0, 1, 2 and 3+). The overall response of students to closed questions in each flag group was tabulated. Open questions were analysed using an approach that allowed the generation of initial themes through an inductive process; each author independently evaluated the data, the primary thematic categories and subthemes were agreed and then analysed. Quotes were selected in relation to the whole dataset. This nested mixed method ( Creswell, 2003; Lieberman, 2005) facilitated rapid data analysis that allowed a combination of quantitative analysis of the large open question dataset with the in-depth investigation of the answers embedded in the survey.

## Results/Analysis

Qualitative analysis identified 3 over-arching themes (‘Before University’, ‘Transition’ and ‘at University’) which are discussed in relation to the underlying sub-themes.

### 1. Demographics

**Figure 1:**
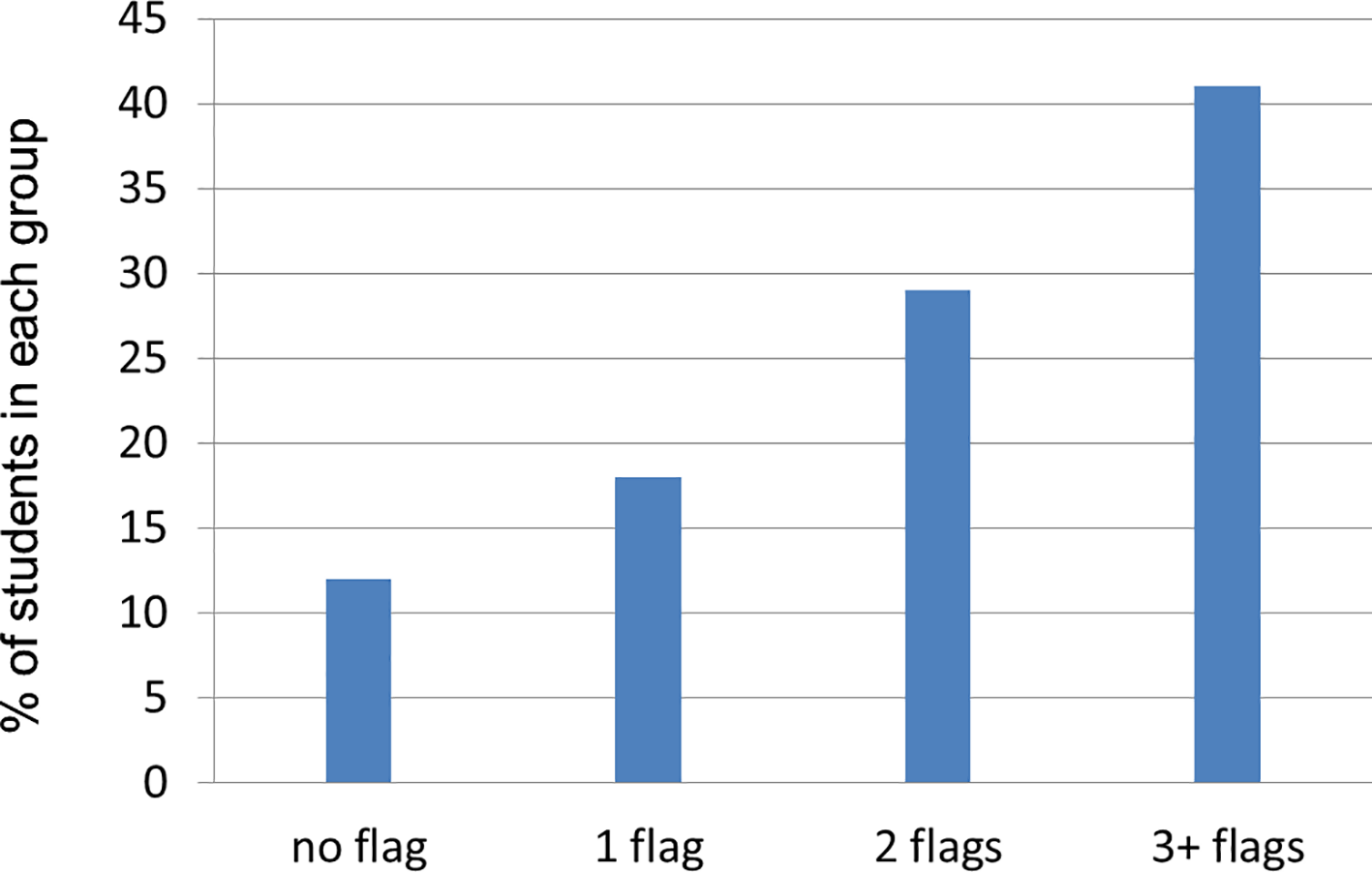
Percentage of students in each group based on the number of WP flags assigned.

24% of the sample were formerly in receipt of free school meals or maintenance grants, 54% were from deprived areas, based on POLAR and/or SIMD and 31% had one or both parents without HE qualification ( Table 1). Most had attended schools with low HE participation rates (LPS) or had come from Further Education colleges. Figure 1 shows sample distribution according to the number of WP flags assigned.

**Table 1:** Demographics of the study sample.

total: 125	SIMD20/40/POLAR3 Q1&2	FSM/EMA	1 ^st^ in family	Attended LPS
n =	67	30	39	79

### 2. Before University

Participants answered a series of questions about their secondary school educational setting, as well as the types of support they received from their schools, outreach initiatives and family members.

The proportion of students that had attended WP outreach programmes increases with increasing number of flags. 50% of students with ***1 flag***, 63% of students with ***2 flags*** and 78% of students with ***3+ flags*** had attended outreach programmes, whereas only 7% of students with ***0* flags** had attended such programmes.

#### 2.1 Family Support.

**Figure 2:**
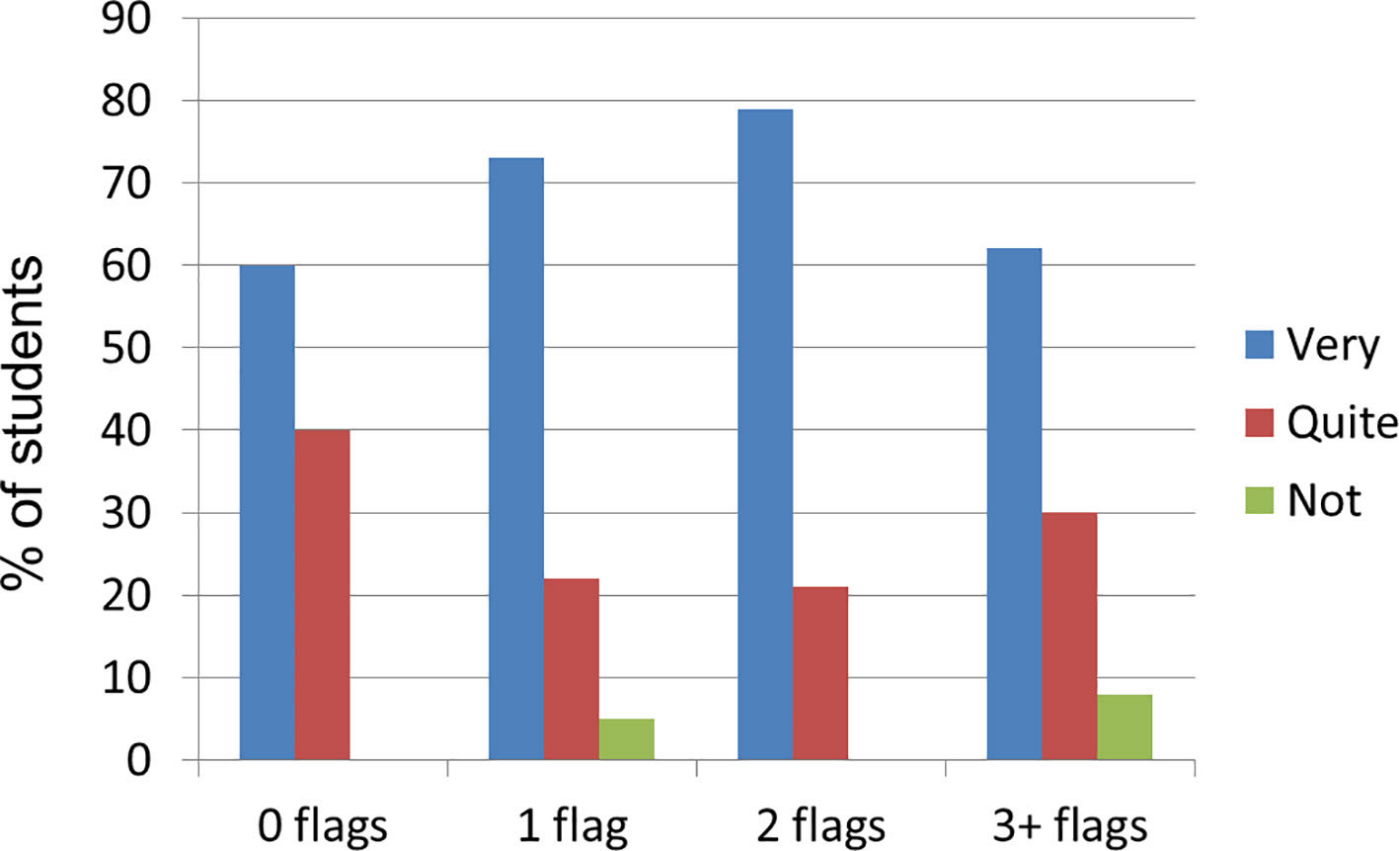
The extent of support students perceived that they had from their immediate family.

Most students, irrespective of the number of flags, said their parents were very supportive (range between 60% and 79%; Figure 2).

However, the qualitative data revealed that the types of support given varied according to the student’s background.

Students ***with 0 flags*** generally received the most comprehensive types of support and the families had the means to cover all forms of support needed: academic, financial, help with application, help with sourcing work experience. Financial support for this group of students appears to be sustained over years (e.g., private school fees or private tutoring). This group had very few comments about receiving emotional support.

*‘Very supportive. Paid for private school, helped with application and work experience contacts’ [P51, 0 flag]*

***Students with 1 flag*** also mentioned most forms of support. However, unlike those with *0 flags* the support was fragmented instead of the ‘complete package’.

*‘I’d like to say that my sister who studies nursing has been of great help as she aided me throughout the application at times; as no one in my family has a job in the healthcare field, she was the only person to be able to help me’. [P123, 1 flag]*

Some families of such students express hesitance concerning an ambition to study medicine. These students also talked about receiving moral support. Neither issue was mentioned by students with ***0*** flags.

*‘Supportive in my education, hesitant towards med[icine] as thought it was too hard/competitive’ [P78, 1 flag]*

These students needed to go outside their immediate family for support and begin to display missing capital, specifically social capital, indicative of a dearth of the comprehensive support that is the norm for students from the least disadvantaged group.

*‘Very. Uncle a doctor so provided work experience. Otherwise just emotionally supportive’ [P117, 1 flag]*

*‘Very happy with my decision, however don’t come from any background from medicine so weren’t able to provide support with my application’ [P92, 1 flag]*

Financial support, sometimes substantial, was also evident; logistic support is now mentioned, for example being driven to Open Days.

Support for ***students with 2 flags*** was further fragmented. Their parents are able to provide only some forms of support; most mentioned are emotional and pastoral support. Single aspects, rather than multiple forms of support are more frequent in this group. Some students still show evidence of using social capital but this is sporadic and often indirect. Interestingly, the qualitative data also shows that, in this category, the support received is ‘diluted’ compared to ***0/1 flag.*** For example, academic support from parents is ‘giving time to study’ rather than direct aid with school work. Emotional supportand encouragement, however, is generally evident **.**

*‘My parents have been very supportive since my decision to apply to medicine, and throughout university so far. The support is mostly emotional though!’ [P53, 2 flags]*

Support in making the application in this group is rarely referred to and when it is, it is more basic, such as reading personal statements, or logistics support for travelling to interviews, but there are frequent mentions of not having money.

*‘Have been very supportive e.g. took me to interview, read my personal statement’ [P91, 2 flags]*

*Students **with 3+ flags*** received very little support linked to the application process due to lack of ‘privileged’ knowledge of this and the course itself. These students clearly lacked the connections provided through social capital that would have helped them with the application process.

*‘Supportive in their encouragement but had no real ability to help me with UKCAT/UCAS etc. Didn’t really know how it all worked.’ [P40, 3+ flags]*

These students also reported more **‘**emotional’ and even reported ‘passive support’ such as notinterfering.

*‘Very supportive and not “pushy”. I came to the decision to apply to medical school through what I had been exposed to in school and predominantly through the [redacted outreach] programme. My parents were patient and did not interfere with my studies/choices in any way’. [P66, 3+ flags]*

However, some in this group experienced discouragement concerning Medicine although there was stillencouragement of ambition.

*‘Would have preferred I studied something else but got behind me after I made my decision’ [P77, 3+ flags]*

There were no quotes about help with academic work from their families, but financial supportwas mentioned, albeit in the context of small contributionssuch as books. This group of studentsoften talked about finance in terms of ‘lackof’ **,** and ‘sacrifice’ **.** Quotes are more about what is missing rather than what was available.

*‘My parents have offered me both financial and emotional support and are prepared to make sacrifices in order to support my education’. [P86, 3+ flags]*

In summary, students with no flag appeared to have the entire range of support but did not mention emotional support. This was also rarely mentioned by students with ***1 flag***, who still received a good deal of other forms of support. Support was increasingly fragmented for those with ***2*** or ***3 flags*** **,** while mentions of emotional encouragement increase as do mentions of logistical help.

#### 2.2 School Type, Support and Academic Achievement

The respondents attended a mix of state, independent and selective grammar schools, with a clear distribution by number of flags ( Table 2).

**Table 2:** School types attended by the student groups (%).

School type	0 flags	1 flag	2 flags	3/3+ flags
State	69	89	92	100
Independent	23	11	3	0
Selective grammar *	8	0	5	0
% total	100	100	100	100

* Grammar schools are government-funded secondary schools, selecting pupils on the basis of their academic ability at age 11; they focus on academic studies, with the assumption that many of their pupils would go on to Higher Education

**Figure 3:**
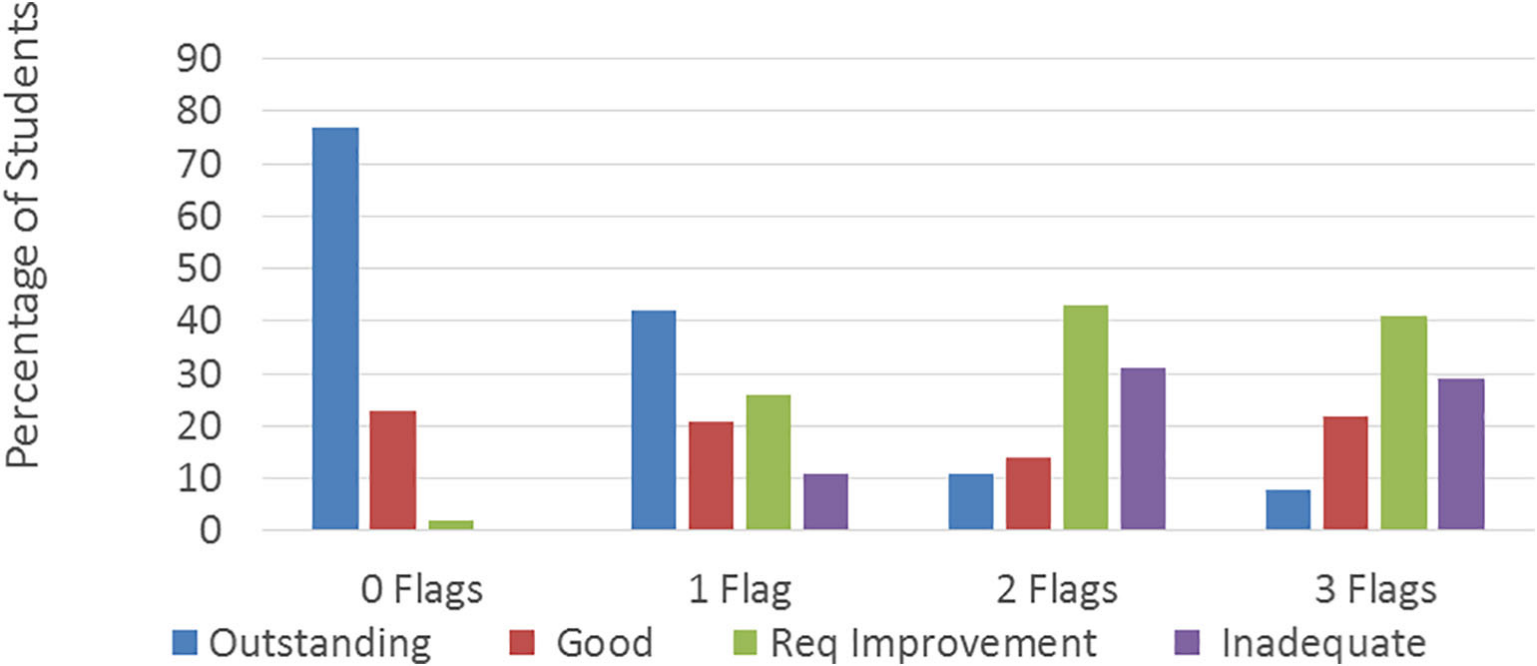
Relationship between number of flags and performance of the school attended.

Students with ***no flags*** are much more likely (77%) to attend schools that are rated ‘outstanding’ by Ofsted (the UK regulator of Secondary/High schools) than those with three or more flags (8%).Conversely, 70% of students with ***3+ flags*** attended schools that are rated as ‘requiring improvement’, or ‘inadequate’.No students with ***0 flags*** attended ‘inadequate’ schools and only 2% attended schools that ‘require improvement’. Thus, there isa clear relationship between the quality of the school attended and number of flags of its pupils ( Figure 3), clearly illustrating the inequality of access to secondary education across the groups. It is not a stretch to say that the type and performance of the school attended likely influenced student’s chances of accessing a Medical Degree.

#### 2.3 Support for Application from School.

**Figure 4:**
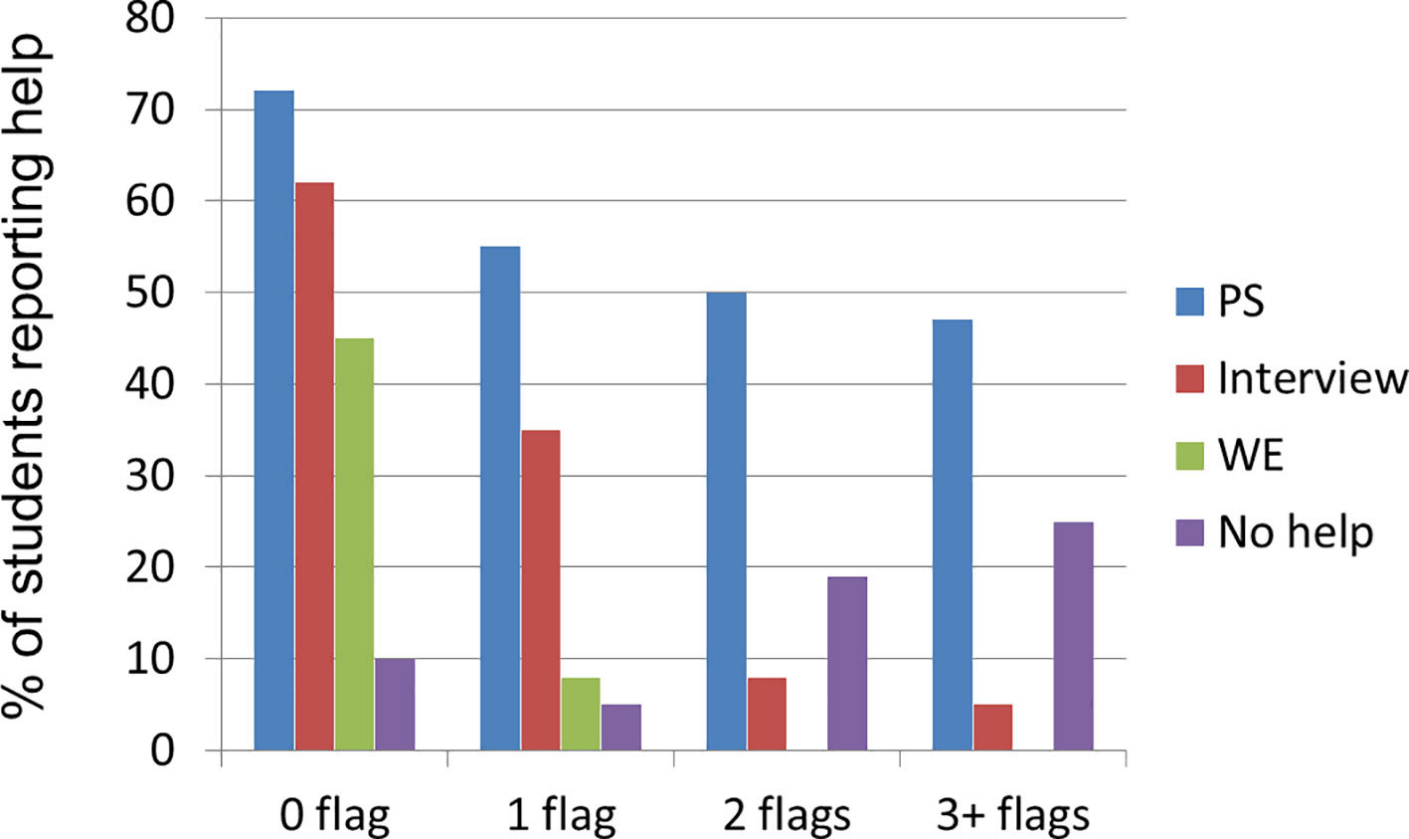
Support given by secondary schools for application to Medical School.

Participants were asked about the types of support they received from their schools when applying to do Medicine ( Figure 4). Students from the most advantaged background had the most support from their schools across all the types of assistance needed (with personal statement (PS); interview; work experience (WE)). Students from the least advantaged background also had the least help; 25% of these students had no help at all.

The most common form of support, irrespective of background, was help with personal statement writing.

The nature of the help provided varies considerably across the demographics: similar to the pattern of parental support, school support becomes more fragmented as the flag number increases and this is most obvious with finding work experience; students with ***2+ flags*** miss out most on this opportunity.

Many pupilswith ***0 or 1 flag*** had very structured, extensive support from their schools, some to the extent that there was a weekly school ‘MedSoc’, and also received by far the most support with work experience.

*‘Helped with personal statements and interview skills. Provided work experience opportunities.’ [P97, 0 flag]*

*‘Attended ‘MedSoc Wednesday nights for aspiring medics.’ [P51, 1 flag]*

Studentswith ***2 flags*** attended schools that providedless structured support that appeared to be on an ‘ad hoc’ rather than institutional basis. Work experience was not available due to lack of relevant connections (social capital). Some students reported that their teachers actively discouraged or even obstructed them.

*‘In fact, we were discouraged to do medicine and apply for biomed instead.’[P70, 2 flags]*

*‘No support for UKCAT (to the extent that I was not given permission from my head teacher to take the morning off to sit it) and no support with interview techniques.’ [P18, 2 flags]*

Incidentally, some pupils in this group reported evidence of a ‘Missing Middle’, whereby they were not quite disadvantaged enough to qualify for special opportunities but definitely not advantaged either:

*‘I was not eligible for the work experience opportunities sent out for medicine as I didn’t ‘tick all the boxes’. I found that if you knew somebody you were able to get work experience... but if you were trying to get it through school/ schemes advertised at school, they were for individuals who met all of the WAMS criteria.’ [P94, 2 flags]*

Studentswith ***3+ flags*** generally experienced a’do it yourself’attitude from their schools, but also provided examples of help from individual teachers going the extra mile to support them.

*‘I had an excellent head of year who was very supportive...he put in a lot of effort with me when preparing my UCAS application, but aside from that my school has a very low rate for people applying to medical school so this was not a territory they were very familiar with.’ [P77, 3+ flags]*

There was a lack of knowledge about application in schools attended by the ***3+ flag*** students. No ***3+ flag*** students received help with work experience and they were least likely to get help at all ( Figure 4).

*‘My guidance teacher didn’t know what a UKCAT was and didn’t know that I would have to do work experience.’ [P23, 3+ flags]*

### 3. Transition

A majority of the participants responding to this question reported that on arrival they felt unprepared for transition from school to university (49/86); these were predominantly from a WP background (43/86). Only 10% of students with ***no*** WP flags felt unprepared for university, while the number more than doubled (23%) for those with *one* or *more* WP flags).

#### 3.1 Preparedness

When asked in what way they felt unprepared, students cited deficient knowledge, a lack of peer support, feelings of isolation and difficulties adjusting to a new way of learning.

They also felt a lack of comparable knowledge, particularly with the postgraduate students or those from the higher-ranking schools.

Those without previous experience of higher education lacked confidence and felt that the expectations at university were unsettlingly different from what they knew from schools. Many comments were made about group work with postgraduate students being intimidating, and how this discouraged their equal participation in the group.

*‘It was very difficult to feel like I deserved my place as it was very easy to start comparing myself to postgraduates or privately taught students who had more knowledge and was therefore better prepared, it was very easy to feel intimidated by the postgraduates initially.’ [P28, 2 flags]*

In contrast, students believed that working with students from their own background provides a network that is important for their learning and emotional transition:

*‘Was so much more different than expected, much more lonely than high school so was a difficult emotional transition.’ [P87, 2 flags]*

*‘Socially [it was difficult] with regards to lack of peers from similar backgrounds, felt unprepared for this as I didn’t expect to be one of very few.’[P45, 3+ flags]*

Students reported difficulties adapting to a new way of learning, particularly around time management, self-directed learning and note taking skills during lectures. Students with 1 or more flags found it hard to self-motivate and felt that their schools didn’t prepare them for university.

*‘Coming from a poor state secondary school, my entire approach to exams consisted of doing past papers and learning answers. When I arrived at medical school, it was assumed that I would know how to study and how I learned best. PBL was an absolute nightmare because of this’. [P53, 1 flag]*

*‘I was completely unprepared... I had zero knowledge on how to manage my time effectively (this was not even helped by going to visit my student adviser or [name of a learning adviser] because I don’t think they understood that I had no idea how to ‘study’) and I didn’t know how to manage the work load; ...my school doesn’t have a high rate of students who go onto professional degrees like medicine so i wasn’t able to get in touch with someone e.g. from the year above me at school who was doing medicine to get advice. ...I have now learned from my mistakes but would have really appreciated some direction at the start of university’’ [P77, 3+ flags]*

Students entering immediately from schools, especially those with low participation in HE, have not learned how to find and appraise information by themselves:

*‘Appraising scientific papers - [have] no experience from school on this. Academic reading was so so difficult because we hadn’t any experience’ [P78, 3 flags]*

Some of the responses described a difficult and emotional transition period, and the pressure of expectations to fit in, having to adapt too quickly:

*‘In most ways, the way of teaching was vastly different (obviously); what I found hardest was having to learn on your own and take notes; student life and the expectation to fit in! [P6, 0 flags]*

### 4. Student Perception related to background

**Figure 5:**
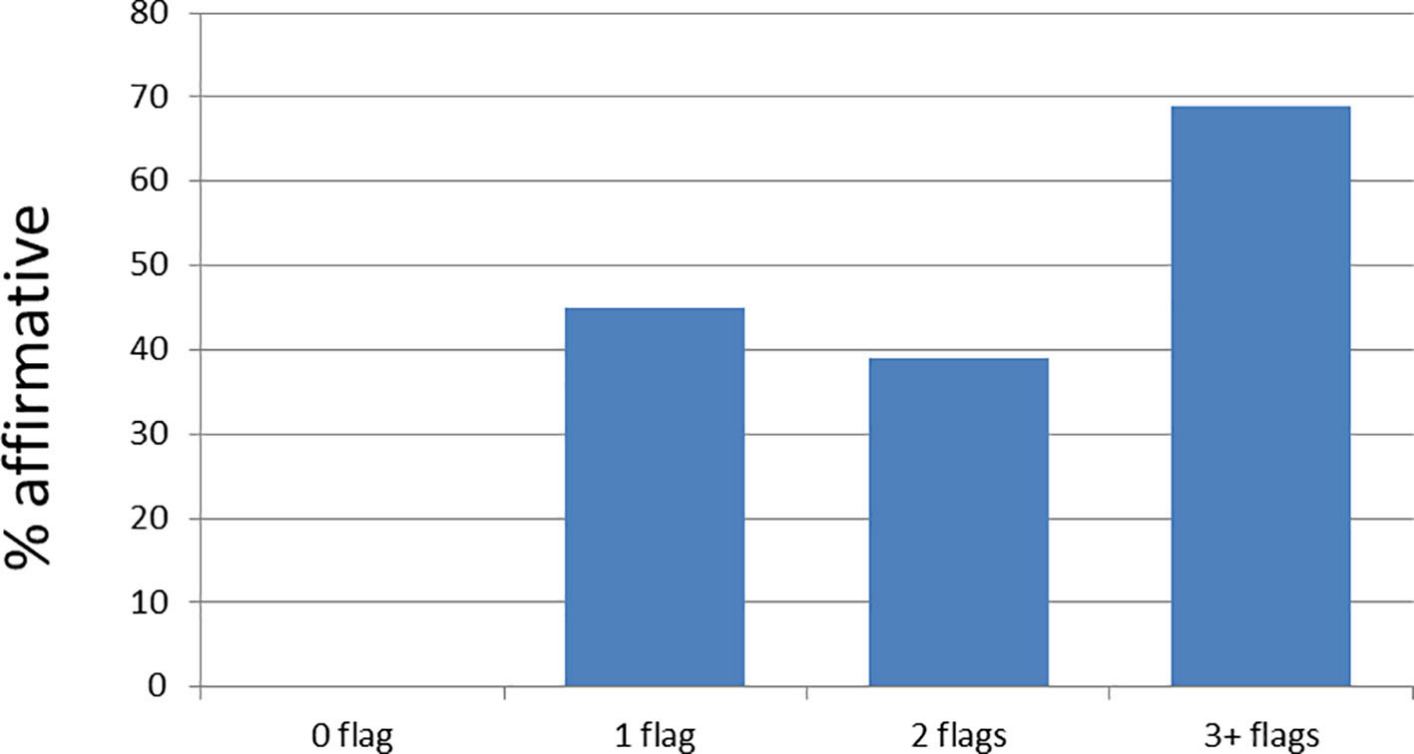
Perception of background disadvantage versus number of flags

53% of those who responded to this question felt that their background had not disadvantaged them in any way when in medical school, while 47% felt that it did, though the response pattern changes with the number of WP flags ( Figure 5).

Finances and lack of confidence in their academic abilities were named as the main reasons for feeling disadvantaged.

*‘Can’t afford all the fancy study guides or text books. Can’t afford to join sport teams or societies, so less involved with year group.’[P15, 3+ flags]*

*‘Others have come more prepared academically than me’ [P93, 2 flags]*

The quotes demonstrate a lack of appropriate economic capital. They all refer to financial restraints that limited their achievements and competitiveness.

Attending the schools they did, the students with ***1 or more*** WP flags felt that their knowledge from school and their social capital, too, are inferior to the ones pupils from private schools have:

*‘Many other students have parents who are doctors or work in the medical profession, or went to private schools. I get the impression that they get a lot of support and advice from parents, and those who went to private schools had exceptional opportunities for extracurriculars. Before university I didn’t know anyone who was a doctor and so have never had someone to ask for specific advice.’ [P113, 1 flag]*

Those who responded negatively to the question felt grateful that they are even there, against the odds:

*‘I think my background has enabled me to be more grateful and driven to make the most of the opportunity I have been given with my place at university.’ [P109, 3 flags]*

### 5. Academic Achievement at Medical School

**Figure 6:**
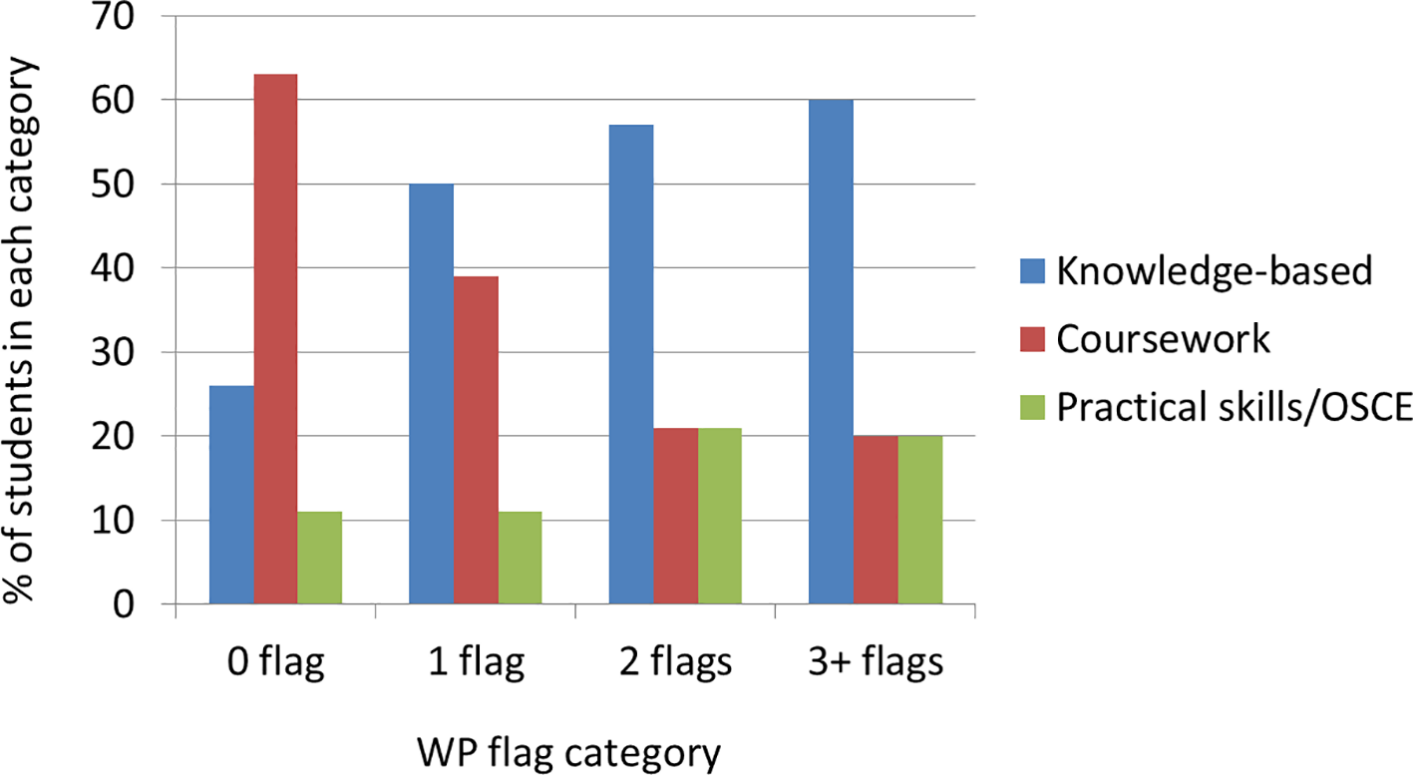
Most difficult assessment - Comparison between the students with varied number of flags.

We asked students which types of assessment they found most challenging, asking them to choose between knowledge-based examinations (written papers), coursework (e.g. essays and other in-course assessment) and practical examinations (OSCE). There was a reasonably even response rate between the groups of students with different numbers of WP flags ( ***0 flags*** 22%; ***1 flag*** 33%; ***2 flags*** 16%, ***3+ flags*** 29%). Among the students with one or more WP flags, over 50% found knowledge-based examinations the most challenging, while 11% ( ***0 flags***) to 20% ( ***3+ flags***) of students considered OSCE the hardest exam ( Figure 6).

Their comments included finding studying for examinations isolating, and highlighted the disparity in knowledge required for university versus school assessment; the time needed for exam preparation was also mentioned:

**‘I find that I have to spread myself quite thin if I want to have a balanced lifestyle and still do well in exams.*’ *[P28, **2 flags**]**

In contrast for students with ***no WP flags***, coursework was deemed the most challenging assessment by 63% (12/19) of students:

**‘It is very difficult to know what to write about and to arrange the writing in an organised way which fits all the marking criteria while trying not to make it all up* *.*’ *[P102, **0 flags**]**

**Figure 7:**
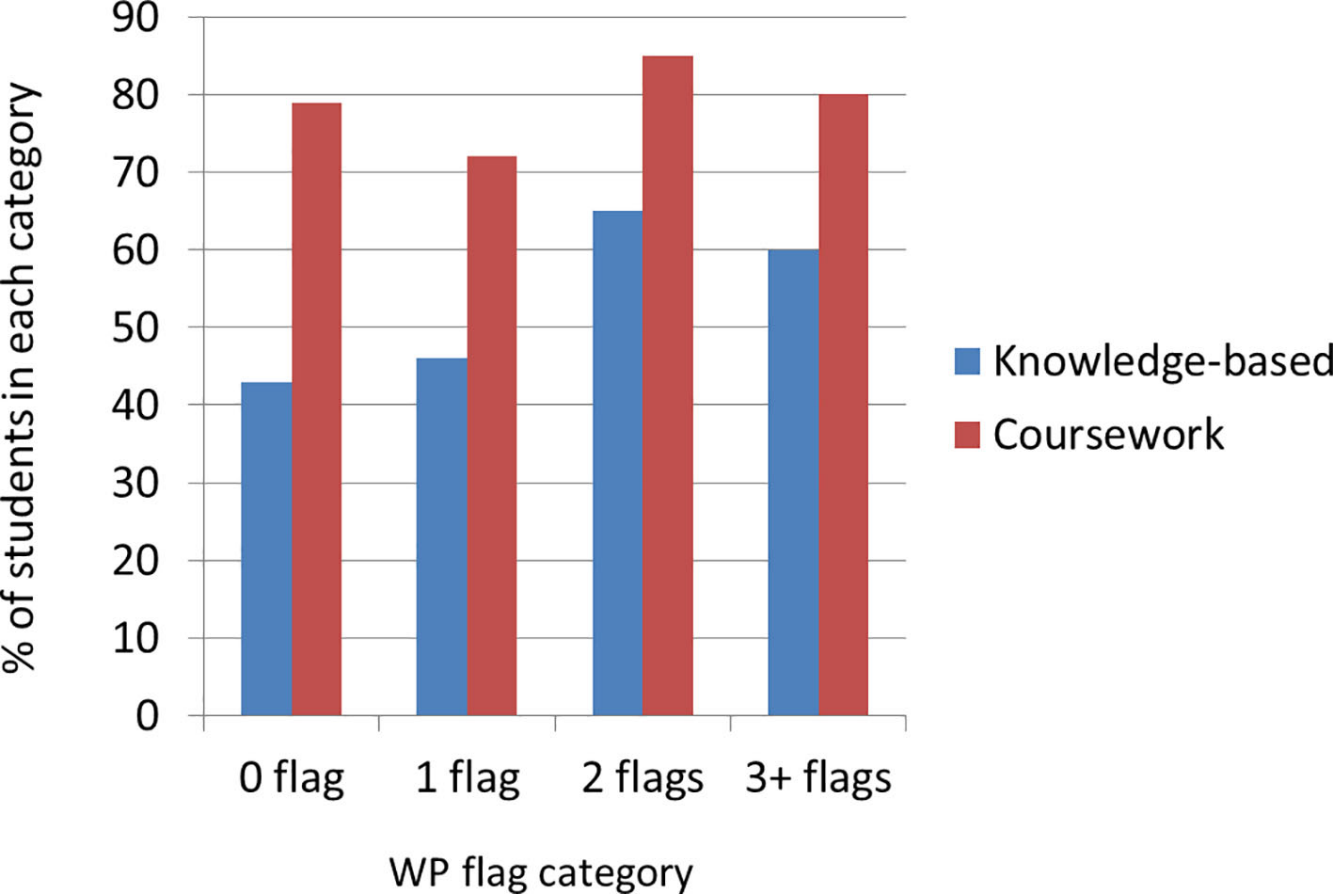
Perception of grade adequacy for students with different number of WP flags.

When asked whether they felt their examination grades accurately reflected their academic ability, for students with ***no*** or ***one* *WP flag*** (n=38), a minority (~40%) agreed they did. In contrast, 60% of students with ***2 or more WP flags*** (n=45) believed that their examination grades reflected their academic ability ( Figure 7).

Student comments relating to this question show there is more anxiety from students with fewer WP flags, related to exams and achievement:

**‘I have not been someone to fail in the past.*’ *[P67, **1 flag**]**

Students with ***two or more flags*** felt that although their grades reflected their ability, they had to work harder for them:

*‘Being from a [outreach] school you sometimes feel ‘imposter syndrome’ and that you are putting in a lot of work compared to other people.’ [P1, 3+ flags]*

As for the coursework assessment, there was consensus across the board that the grades reflected their academic writing ability. However, WP students felt that others had more access to support, for example from parents.

*‘... I have had to rely solely on myself for this academic writing. I feel that a lot of students can send their essays to their parents for a last check, but I don’t have that and must rely on my own self.’ [P122, 3+ flags]*

### 6. Academic Support at Medical School

The types of learning support that WP students would like to access were the same for all groups, e.g. note-taking, reflective writing, academic and scientific writing, time management and critical thinking, with many students noting that these skills were key to successful transition to university.

The majority of students, irrespective of WP flag (62-85%) felt that their medical school had supported them well academically with course content, but over half of all groups (57-62%) felt they would have benefited from extra academic support in addition to the generic skills mentioned above:

*‘Extra sessions for people who are struggling a little with the workload e.g. extra lectures for the basic anatomy or physiology’ [P105, 1 flag]*

*‘I think one-to-one sessions with an academic should be given for half-an-hour to an hour for widening participation students each semester or so to help clarify anything’. [P122, 2 Flags]*

## Discussion

This study attempts to understand students’ perception of barriers to accessing medical studies, the support they get from schools and families in order to enter medical school and their ability to integrate with the student community and progress on the course. The responses of students with varied number of WP flags reveal differences in (the perceptions of) these challenges, and themes that either bestowed advantage or disadvantage in overcoming them.

Applying Bourdieu’s different forms of capital, our analysis revealed important trends as the three primary forms of capital accrued by the students influenced their journey to a medical education. The importance of economic capital (wealth and income) was manifest in the ability to pay, for example, for private schooling, tutors, preparation courses, Open Day visits, ‘fancy study guides and text books’, and was primarily the purview of those with fewer flags. Giving a sense that for students with fewer WP flags their ‘economic capital’ helped them to achieve the necessary academic requirements. School type is another aspect of economic capital that appears to be a key way in which advantages and disadvantages are perpetuated for students from different backgrounds. Those who attended ‘better’ schools were given structured, dedicated support within their school, not only to attain the high grades required, but also to navigate the selection process, which was not available in most schools with low HE participation rates. In the same way the ‘social capital’ (valuable connections) accrued by those from more advantaged backgrounds is manifest in forms of ‘privileged’ knowledge of the ‘system’. This includes obtaining work experience and help from parents, relatives or family friends in the medical professions. Those from less privileged backgrounds did not have such links and often relied on, for example, one helpful teacher. Interestingly, people with parents who are doctors are 24 times more likely to also become doctors, than those whose parents are not. This social reproduction ( Jonsson *et al.,* 2009) in the medical professions highlights the importance of insider information and work experience, to gain admission to the medical school. The mismatch of cultural capital is more difficult to ascertain with this survey. Cultural capital alludes to ‘symbolic mastery’, which manifests in the use of elaborative vocabulary, correct grammar, cultural cues, activities and tastes. The matching/mismatching of cultural capital may play out in selection interviews (by interviewers with matched cultural capital), and impacts strongly on the feeling of ‘fitting in’ at Medical school - an important theme in the returns from WP students. They often emphasised a lack of integration and peer support during the transition.

Feelings of ‘not fitting in’ for WP students transitioning into the medical school, are exacerbated by their lack of economic capital, which often excludes them from many social, leisure and sports activities. In many cases these students have to work at weekends/evenings for extra income. This exclusion impacts on their ability to participate in (conversations about) key activities, leading to a perpetuation of their deficit in terms of social and cultural capital throughout their time in the medical school.

Many of the issues identified in this study, such as lack of privileged knowledge, change in learning approach and lack of peer support, are difficult to overcome but can certainly be ameliorated by early outreach programmes, initial learning support and managing expectations at entry into the medical school. Managing expectations is a prerequisite for students’ success and progression on the course ( Brinkworth *et al.,* 2009; Kandiko and Mawer, 2013), as it gives structure to their learning process and, crucially, reduces the performance-related and social anxieties that are especially high during transition, when students have not yet built a support network. The social integration could be improved by non-academic group activities early in the first year, and by the encouragements of peer support structures for WP students, including mentoring schemes.

## Conclusion

The work presented here lays out the differences in perceived and actual barriers for students with different number of WP flags in three medical schools. These barriers go far beyond the difficulties of achieving the requisite top grades in under-performing schools. Nor are the barriers limited to the application and admissions process: even after admission, mismatching social, economic and cultural capital continue to make the transition and progression on the course more challenging for WP students. Background and, specifically, the social ‘class’ of prospective students affects not only access but the attainment of even those WP students that do successfully gain admission to medical school.

It is clear from the study that the process of admission and the difficulties of transition contribute to the preservation of social stratification in Medicine, resulting in it remaining one of the most closed-off professions to those from lower socio-economic classes ( Friedman and Lauriston, 2019). Despite the hopes of policy makers, the effects of class background persist as a barrier or enabler for access and progression in the medical profession.

## Take Home Messages

• Students from lower economic and social classes remain highly underrepresented in the medical profession and the barriers for these students go far beyond the challenges of achieving the required school grades.
• Irrespective of the number of WP flags, current medical school students felt supported by their families to pursue medicine but the type of support given differed with the student’s economic, social and cultural capital.
• Structured support towards the application and selection process was only available at schools with high HE participation rates.
• WP students found transition from school to university challenging, citing a lack of knowledge, changes in learning style and a lack of peer support.
• Interventions such as initial learning support and managing expectations in the early years at medical school will aid a successful integration.

## Notes On Contributors

Nana Sartania, Louise Alldridge and Clare Ray are all members of the National Medical Schools Widening Participation Forum.

**Nana Sartania** is Deputy Director of Admissions and Senior Lecturer in the Undergraduate Medical School in University of Glasgow. Her research interests are concerned with the predictive validity of the admissions tests used in the UK and the use of contextual data in the admissions process to support diversity.

**Louise Alldridge** is Associate Professor in Medical Sciences and Lead for Widening Participation and Programme Lead for BMBS with Foundation Year in Peninsula Medical school. Her research interests include inclusivity and equality in medical education.

**Clare Ray** is a Reader in Widening Participation in Biomedical Education and is the College lead for Outreach and Widening Participation at the University of Birmingham. She is the academic lead for widening access to medicine activities and a programme of peer mentoring and professional development support for students entering via WP route.
