# Supplementary material for: Barriers to access, transition and progression of Widening Participation students in UK Medical Schools: The students’ perspective
Source: MedEdPublish (2016). 2021 May 17;10:132. [Version 1] doi: 10.15694/mep.2021.000132.1 (PMC10939539; doi:10.15694/mep.2021.000132.1)
Supplement: Peer review history

# Reviewer report: Reviewer response for version 1

**DOI:** 10.21956/mep.18944.r27104

**Published:** 2021-06-18

**Authors:** Enam Haque

**Affiliations:** 1 University of Manchester

This review has been migrated. The reviewer awarded 5 stars out of 5 Widening participation is key to Medicine, if we are to have doctors that are representative of the population they serve. There has been excellent progress, through the work of the Medical Schools Council Selection Alliance and National Medical Schools Widening Participation Forum. However, the inequalities facing applicants and medical students still exist. This pansector study clearly lays out the issues, and opens up discussions on what medical schools need to do, in order to address student access, success and progression issues. Well done to the authors on producing this excellent paper.

Reviewer Expertise:

NA

No decision status is available

# Reviewer report: Reviewer response for version 1

**DOI:** 10.21956/mep.18944.r27105

**Published:** 2021-06-02

**Authors:** John Cookson

**Affiliations:** 1 University of Worcester

This review has been migrated. The reviewer awarded 5 stars out of 5 Thank you. An interesting and important contribution and the use of the number of 'flags' as a measure is innovative.My only problem was that I couldn't find the denominator for the number of respondents. It looks as if the research sample was a sub group of a sub group, so generalising the findings could be difficult.

Reviewer Expertise:

NA

No decision status is available
